# Predicting symptom response and engagement in a digital intervention among individuals with schizophrenia and related psychoses

**DOI:** 10.3389/fpsyt.2022.807116

**Published:** 2022-08-11

**Authors:** George D. Price, Michael V. Heinz, Matthew D. Nemesure, Jason McFadden, Nicholas C. Jacobson

**Affiliations:** ^1^Center for Technology and Behavioral Health, Geisel School of Medicine, Dartmouth College, Lebanon, NH, United States; ^2^Quantitative Biomedical Sciences Program, Geisel School of Medicine, Dartmouth College, Lebanon, NH, United States; ^3^Department of Psychiatry, Geisel School of Medicine, Dartmouth College, Lebanon, NH, United States; ^4^Dartmouth College, Lebanon, NH, United States; ^5^Department of Biomedical Data Science, Geisel School of Medicine, Dartmouth College, Lebanon, NH, United States

**Keywords:** schizophrenia, digital intervention, machine learning, intervention engagement, qualitative impressions, app use, Brief Symptom Inventory

## Abstract

**Introduction:**

Despite existing work examining the effectiveness of smartphone digital interventions for schizophrenia at the group level, response to digital treatments is highly variable and requires more research to determine which persons are most likely to benefit from a digital intervention.

**Materials and methods:**

The current work utilized data from an open trial of patients with psychosis (*N* = 38), primarily schizophrenia spectrum disorders, who were treated with a psychosocial intervention using a smartphone app over a one-month period. Using an ensemble of machine learning models, pre-intervention data, app use data, and semi-structured interview data were utilized to predict response to change in symptom scores, engagement patterns, and qualitative impressions of the app.

**Results:**

Machine learning models were capable of moderately (*r* = 0.32–0.39, R^2^ = 0.10–0.16, MAE*_*norm*_* = 0.13–0.29) predicting interaction and experience with the app, as well as changes in psychosis-related psychopathology.

**Conclusion:**

The results suggest that individual smartphone digital intervention engagement is heterogeneous, and symptom-specific baseline data may be predictive of increased engagement and positive qualitative impressions of digital intervention in patients with psychosis. Taken together, interrogating individual response to and engagement with digital-based intervention with machine learning provides increased insight to otherwise ignored nuances of treatment response.

## Introduction

Schizophrenia, a primary psychotic disorder, is a serious mental illness characterized by debilitating symptoms including delusions, hallucinations, disorganized speech and behavior, and diminished emotional expression (1, 2). Current studies estimate that schizophrenia affects up to 0.64% of the United States population (3–5). However, despite schizophrenia’s comparatively low incidence (6), it had an economic burden of $155.7 billion in 2013 (7), and remains a major contributor to the global burden of disease with two-thirds of affected individuals experiencing persistent symptoms following treatment (1, 8). Further, schizophrenia is often comorbid with anxiety disorders and depression (9, 10). Thus, illness burden is of particular concern, with symptom severity shown to be negatively correlated with physical health, psychological health, and relationships, and one in ten completing suicide (11, 12). Despite recent improvements in diagnostic accuracy and treatment efficacy, schizophrenia continues to negatively impact patients’ overall quality of life (11), highlighting the necessity for additional efforts to address treatment response at the individual level.

Currently, the mainstay treatment for schizophrenia includes the use of antipsychotics, often coupled with regular psychosocial interventions (13, 14). Psychosocial interventions can be offered as individual (supportive counseling, personal therapy, social skills therapy, vocational rehabilitation therapy); cognitive-behavioral; and group (interactive/social therapy) interventions (14). Despite the relative efficacy of these combined approaches, the side-effects of antipsychotic treatment can prove debilitating to schizophrenia patients. Antipsychotic side-effects may include physical symptoms, such as movement disturbances, metabolic derangements and weight gain, sedation, and drooling (15, 16, 17–19), as well as emotional and cognitive blunting (20). As a result, the integration of alternative interventions may prove useful in reducing the side-effect burden of antipsychotics.

Research has shown promise in the use of scalable digital therapeutics in patients living with serious mental disorders, such as schizophrenia and bipolar disorder (21). Although few studies have directly analyzed the efficacy of digital interventions for schizophrenia, existing research has suggested that this method of intervention may be efficacious for schizophrenia treatment or management. For example, PRIME, or Personalized Real-time Intervention for Motivational Enhancement, is a mobile application designed to supplement antipsychotic and psychotherapeutic treatments for schizophrenia. The intervention was found to be effective in improving the mood and motivation of young patients with schizophrenia. Further, PRIME users experienced improvements in depression, self-efficacy, and reward learning (22, 23). Similarly, App4Independence (A4i), a community-centric app designed for schizophrenia patients, offers forums, appointment scheduling, and text-based functions aimed at improving illness self-management (24). Like PRIME, A4i showed modest reductions in symptoms of depression, further pronounced after controlling for gender, age, and other baseline symptoms (24).

Treatment of schizophrenia remains a challenge, partially owing to its highly heterogeneous nature (25–29), with little known of personalized prognostic and treatment factors. With growing numbers of digital treatment options for mental health in the context of this heterogeneity, it is important to understand not only the group efficacy of such interventions, but also the profile of those individuals most likely to benefit. With such an understanding, individuals could be effectively “matched” to the specific interventions most likely to improve their symptoms.

Machine learning, coupled with highly dimensional datasets, like A4i, is uniquely positioned to address these challenges, having shown promise in constructing personalized models, with a capacity to accurately predict individual-level treatment response (30, 31). Specific to individuals with schizophrenia, an extensive corpus of research has shown machine learning efficacious in classifying schizophrenia and psychosis-related symptoms from neuroimaging data (32–34), qualitative social media information (35, 36), and passively collected smartphone data. Notably, virtual communication was positively associated with increased negative affect measures (37), highlighting the necessity to interrogate the driving factors in engagement with digital interventions in individuals with schizophrenia. Furthermore, machine learning has the capacity to model complex relationships in large datasets, with model introspection made possible by high power computing methods, such as Shapley Additive Explanations (SHAP) (38). Methods, like SHAP, address the “black box” problem of machine learning by offering a manner of visually representing the directionality and magnitude of those features most important in the model’s outcome prediction. This allows for the development of downstream digital biomarkers and phenotypes of psychiatric disorders.

In the current study, we utilize data from an open trial delivering the A4i intervention to persons with schizophrenia, schizoaffective disorder, and related psychoses that found modest symptom improvement at the group level (24), to better understand personalized markers of digital intervention engagement and response. We hypothesized (1) *unique baseline patient* characteristics paired with machine learning would moderately predict (*r* > 0.3) individual symptom response to, engagement with, and sentiment toward A4i (an app-delivered, digital intervention) (2) specifically, we hypothesized that people with higher affective symptoms (e.g., depression) and lower psychotic symptoms would have the most robust use and response, resulting in more positive sentiment toward the app; this hypothesis can be contextualized in research which suggests better overall prognosis and treatment response for patients with higher affective symptoms (39). Further, (3) we hypothesized that persons with higher interpersonal sensitivity would have the strongest response to the intervention (change in composite BSI score), given the built-in peer-engagement application feature, which was reported in the original study as a user-reported strength of the A4i intervention (24). To evaluate these hypotheses, we developed three machine learning pipelines aimed at modeling the relationship between baseline patient characteristics and (i) response to the digital intervention, (ii) level of engagement with the intervention, and (iii) user sentiment toward the intervention.

## Materials and methods

### Participants

Participants (*N* = 38, 2.6% transgender, 71.1% men, 26.3% women, age*_*mean*_* = 31.42 ± 8.60) were included in the final study population based on previously described inclusion and exclusion criteria (24). The initial study design consisted of a 3–4 week engagement with the A4i app with pre-post assessments. The study was reviewed and approved by an institutional Research Ethics Board; participants provided written consent, and the protocol was registered with clinicaltrials.gov (NCT03649815) (24).

### Intervention

The A4i functionality included personalized prompts, activity scheduling, connections to social engagement resources, evidenced-based content tailored to management of psychotic symptoms, a peer engagement network, daily wellness check-ins, and passively collected phone-use information, used as a proxy for sleep and activity (24).

### Data collection and outcome metrics: Quantitative data set

Participants provided demographic information, mobile technology use information, and completed quantitative pre-post symptom and intervention engagement metrics (24). The quantitative metrics included: (a) the Brief Adherence Rating Scale (BARS) to examine implications of A4i for medication use (40), (b) the Person Recovery Outcome Measure (PROM) to assess degree of engagement in the recovery process (41), and (c) the Brief Symptom Inventory (BSI) to assess psychiatric symptoms pre and post-intervention. The BSI comprises domains measuring: psychoticism, somatization, depression, hostility, phobia, obsessive-compulsive, anxiety, paranoia, and interpersonal sensitivity (42). Additionally, A4i usage data from each participant during the trial was passively collected, including: (i) a count of participants’ total active interaction with the app, (ii) the number of days each participant engaged with the app, (iii) the participants’ average interaction with the app per day, and (iv) app usage categorized as “low” or “high.” Participant BSI total and subdomain scores were included in the present analysis; BARS scores, PROM scores and demographic information were not.

### Post-intervention semi-structured interview

A semi-structured interview was completed at the post-A4i use assessment, which included a series of seven questions providing qualitative feedback from the participants on the functionality, effectiveness, and overall experience interacting with the app (e.g., “What were your favorite features of the app?”) (24). The complete semi-structured interview is provided in the Supporting information section of the original publication (24).

### Semi-structured interview response sentiment quantification

We extracted participants’ overall response sentiment from the semi-structured interviews. Individual responses to all questions were concatenated using Python (v3.8.3) for uniformity across participants (43). Overall response sentiment was derived from the concatenated qualitative data, using the Python package *TextBlob* (Version 0.16.0) to assess polarity (i.e., the valence of the participants responses on a –1 to 1 scale, with a lower score reflecting a more negative statement and a higher score reflecting a more positive statement) (44).

### Data preprocessing

Baseline BSI total score, baseline BSI subcategory scores, and passively collected A4i use metric features included in modeling were individually standardized resulting in a μ = 0 and σ = 1 within that feature. Feature standardization has been shown to increase model efficiency and accuracy in machine learning approaches (45), and provides a consistent value range for features when considering their relative influence on a model’s predictions.

### Theoretical machine learning modeling framework

We implemented a hypothesis driven framework *via* the utilization of three separate ensemble machine learning models to interrogate individual-level factors driving (1) app efficacy, (2) app engagement, and (3) qualitative app impressions (see Table 1). The machine learning models implemented in this study were as follows:

1. *Symptom Severity Change*: Fourteen features, including baseline BSI total score, subcategory scores, and passively collected A4i use metrics were used to predict change in BSI total score (Table 1, Model 1). Change in BSI total score was measured as the difference between baseline BSI total score and post-intervention BSI total score (e.g., a negative change reflects an overall decrease in reported symptoms).
2. *A4i Engagement*: Ten features, including baseline BSI total score and subcategory scores were used to predict a participants’ overall interaction with the A4i app (Table 1, Model 2).
3. *Intervention Impressions*: Ten features, including baseline BSI total score and subdomain scores were used to predict an individual participant’s sentiment toward the intervention (Table 1, Model 3). A participants’ sentiment was represented by the polarity score of their concatenated semi-structured interview responses.

**TABLE 1:** Machine learning model corresponding hypotheses, features and outcomes.

Modeling approach	Model features	Model outcome
Model 1: Symptom Severity Change	Baseline BSI Composite Score (Overall Symptoms), Baseline BSI Anxiety, Baseline BSI Depression, Baseline BSI Hostility, Baseline BSI Interpersonal Sensitivity, Baseline BSI Obsession-Compulsion, Baseline BSI Paranoid Ideation, Baseline BSI Phobic Anxiety, Baseline BSI Psychoticism, Baseline BSI Somatization, Total A4i Interaction, Total Days of A4i Interaction, Binary A4i Use (High/Low), Average A4i Use Per Day	Change in Composite BSI Score
Model 2: A4i Engagement	Baseline BSI Composite Score (Overall Symptoms), Baseline BSI Anxiety, Baseline BSI Depression, Baseline BSI Hostility, Baseline BSI Interpersonal Sensitivity, Baseline BSI Obsession-Compulsion, Baseline BSI Paranoid Ideation, Baseline BSI Phobic Anxiety, Baseline BSI Psychoticism, Baseline BSI Somatization	Total Interaction with A4i
Model 3: Intervention Impressions	Baseline BSI Composite Score (Overall Symptoms), Baseline BSI Anxiety, Baseline BSI Depression, Baseline BSI Hostility, Baseline BSI Interpersonal Sensitivity, Baseline BSI Obsession-Compulsion, Baseline BSI Paranoid Ideation, Baseline BSI Phobic Anxiety, Baseline BSI Psychoticism, Baseline BSI Somatization	Semi-structured Interview Response Sentiment (Polarity)

Input features and predicted outcomes for the three interrogated ensemble models.

### Practical machine learning model framework

All machine learning modeling followed the same nested leave-one-out (LOO) cross-validation framework (46). A nested cross-validation framework in machine learning is efficacious in allowing for unbiased performance estimates, regardless of sample size (47). In this process, one subject was completely held out, while the rest of the subjects were used as part of a simple LOO cross-validation approach to tune the hyperparameters of the model. This process was repeated *N* times so each subject was held out at least once. We used an ensemble approach, whereby distinct machine learning models (i.e., linear models, tree based models, and multilayer perceptrons) were individually trained on the data; an approach which has been shown to consistently outperform base algorithms in mental health disorder related outcomes (48). Predictions from these models were used as inputs to a final “deciding” model, which returned a consensus score. The specific modeling architecture and hyperparameters of the Symptom Severity Change, A4i Engagement, and Intervention Impressions models are provided in Supplementary Table 1. The ensemble pipeline’s predictions were evaluated against the observed values to determine the correlative strength (*r*), the proportion of the variance in the outcome explained by the model’s predictions (R^2^), and the normalized mean absolute error (MAE*_*norm*_*) of the respective ensemble model. The normalized mean absolute error was calculated by dividing the mean absolute error by the range of the observed outcome, offering an outcome-agnostic representation of the model’s mean absolute error.

### Model explainability

We implemented Shapley Additive Explanations (SHAP) to aid in model interpretability by evaluating the top five most influential features in each of the three models. Intuitively, SHAP allows for model introspection by iteratively perturbing the input data and assessing how this affects the output (38). In this way, the process can determine feature importance, as well as the marginal contribution of each independent variable to the predicted outcome at the patient level, represented by an individual values positioning on the x-axis of Figure 2. Using this information, SHAP can estimate relative feature importance, directional relationships between predictors and outcomes, as well as different order interactions between variables.

## Results

### Predictive performance and interpretability: Symptom severity change

**FIGURE 1:**
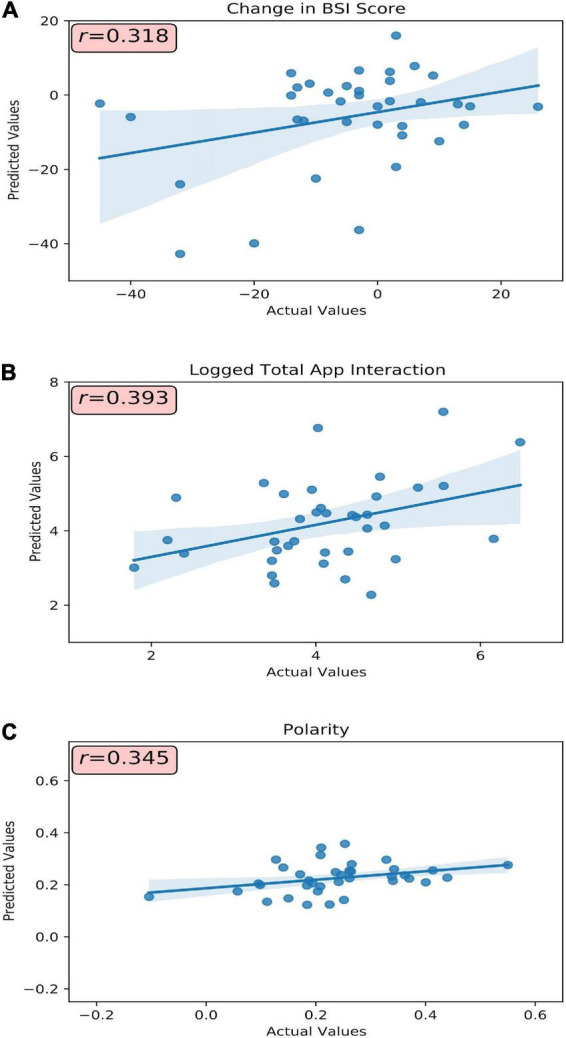
Model(s) actual versus predicted values plotted with respective correlative strength. **(A)** Baseline BSI total score, subcategory scores, and passively collected A4i use metrics were used to predict change in BSI total score. **(B)** Baseline BSI total score and subcategory scores were used to predict a participants’ overall interaction (visualized as the log-transformation) with the A4i app. **(C)** Baseline BSI total score and subdomain scores were used to predict an individual participant’s sentiment toward the intervention. r, Pearson’s correlation coefficient.

**FIGURE 2:**
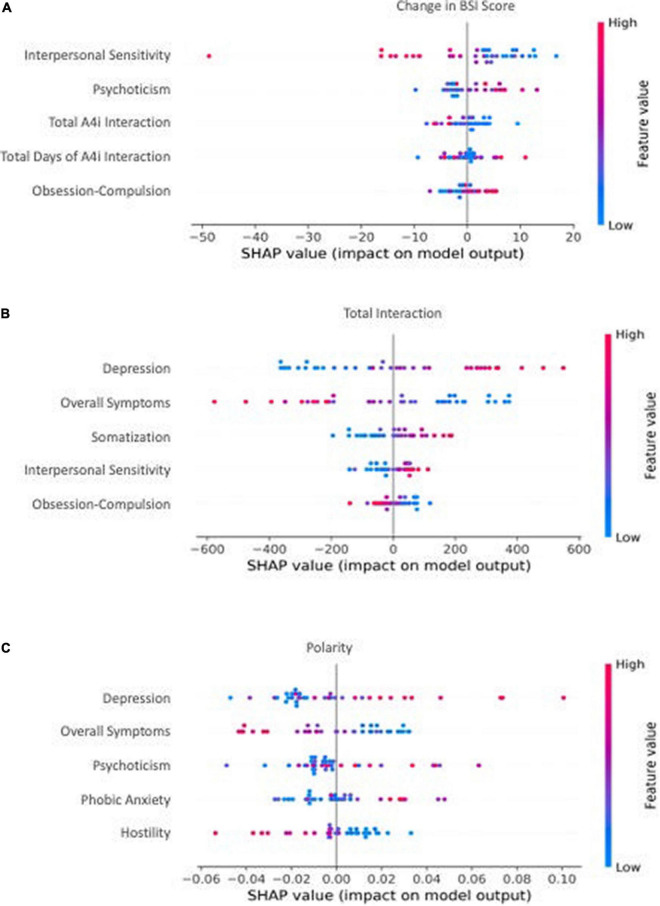
The top 5 most influential features by model. Individual dot color corresponds to the value of the feature, and location on the x-axis corresponds to that point’s relative impact on the model output [e.g., a high-feature value (red) with a corresponding high x-axis value (SHAP value) represents a point that strongly, positively influences the model’s outcome prediction]. **(A)** The most influential features from baseline BSI total score, subcategory scores, and passively collected A4i use metrics for predicting change in BSI total score. A positive x-axis value (SHAP value) corresponds to an increase in overall symptoms. **(B)** The most influential features from baseline BSI total score and subcategory scores for predicting a participants’ overall interaction) with the A4i app. A positive x-axis value (SHAP value) corresponds to an increased interaction with A4i. **(C)** The most influential features from baseline BSI total score and subdomain scores for predicting an individual participant’s sentiment toward the intervention. A positive x-axis value (SHAP value) corresponds to an increase in qualitative A4i impressions.

Participants’ A4i use metrics and baseline BSI scores (Table 1, Model 1) were capable of moderately predicting change in symptom severity (e.g., Pre-Post BSI score difference, where a negative change corresponds to a decrease in BSI score, and thus reduced overall symptoms) (*r* = 0.32, *R*^2^ = 0.10, MAE*_*norm*_* = 0.29) (Figure 1A). Model introspection *via* SHAP suggested that the most influential feature for predicting change in BSI score was *interpersonal sensitivity* on the baseline BSI (Figure 2A), where participants with high baseline interpersonal sensitivity were predicted to have a greater reduction in psychiatric symptomatology. Furthermore, lower psychotic and obsessive compulsive traits were predictive of a reduction in psychiatric symptomatology across the intervention. These findings directly address study hypothesis (1), that unique patient characteristics will moderately predict app response, and more specifically study hypothesis (3), which suggested interpersonal sensitivity as a positive prognostic marker for A4i response.

### Predictive performance and interpretability: App4Independence engagement

Baseline symptom severity (measured by pre-intervention BSI scores) moderately predicted participant engagement across the A4i intervention (*r* = 0.39, *R*^2^ = 0.16, MAE*_*norm*_* = 0.16) (Figure 1B). The BSI subdomain *depression* was the most influential feature, where participants with high baseline depression interacted with the app more during the intervention; however, overall BSI score showed an inverse relationship, where participants with lower overall total symptoms were predicted to have greater app interaction. These findings directly address study hypothesis (1), that that unique patient characteristics will moderately predict person-level app engagement, and more specifically hypothesis (2) that higher affective symptoms would drive A4i response.

### Predictive performance and interpretability: Intervention impressions

Baseline BSI scores moderately predicted valence of interview responses (*r* = 0.34, R^2^ = 0.12, MAE*_*norm*_* = 0.14) (Figure 1C), with BSI depression scores having the greatest importance. Specifically, participants with high baseline *depression* were more positive when discussing the app in the semi-structured interview. Interestingly, similar to the A4i engagement model, overall BSI score showed an inverse relationship to the BSI *depression* subdomain, with lower overall BSI being predictive of more positive qualitative impressions of the intervention. These findings directly address study hypothesis (1), that unique patient characteristics will moderately predict overall app sentiment and, as in section “Predictive performance and interpretability: A4i engagement” – hypothesis (2) – that higher affective symptoms would drive A4i response.

## Discussion

This study demonstrates the capacity of unique patient-level factors to predict response to a digital treatment among patients with schizophrenia, schizoaffective disorder, and related psychoses. Important factors included interpersonal sensitivity, psychotic traits, depressive traits, and overall symptom severity (as determined by baseline BSI), as well as digital intervention interaction metrics passively collected during the study. Subsequent analysis established factors associated with participant interaction, engagement and general attitudes toward the digital therapeutic intervention. As a whole, this work aimed to investigate unique patient markers of digital treatment response, as well as highlight those factors most important in predicting high engagement among persons with psychosis. Our results contribute to ongoing development and implementation of mental health digital interventions by identifying unique patient markers to suggest intervention response as well as engagement.

### Higher interpersonal sensitivity

The *symptom severity change* model (Table 1, Model 1) predicted that participants with high baseline interpersonal sensitivity would benefit more from the A4i intervention. This finding may suggest that high interpersonal sensitivity corresponds to increased participant responsiveness to the community-centric platform of the intervention. The role of patient interaction is highlighted by the users during the semi-structured interviews, with one patient responding that the function of the A4i app is to serve as “a safe space online app based community platform where psychosis can be met with care and empathy.” (24). Thus, in line with findings that lack of interpersonal relationships is known to be a significant contributor to reduced quality of life among individuals with schizophrenia (49), patients that actively interacted with the A4i community responded better to the intervention. Further, these findings are congruent with the A4i intervention goals which sought to target interpersonal aspects of psychotic disorders (24).

A second hypothesis for higher emotional sensitivity predicting better A4i response involves a potential association between interpersonal-affective sensitivity and psychotic symptom disorder severity in persons with psychosis. Interpersonal hypersensitivity has been shown characteristic of prodromal psychosis in clinically high risk patients (50). By contrast, patients at later illness stages show a generalized deficit in affect recognition, not characteristic of their earlier-illness-stage counterparts (51). Moreover, fine recognition of sad and neutral affective states have been inversely correlated with measures of disorganization in schizophrenia (51). Taken together these findings suggest (1) interpersonal and affective sensitivity as a potential surrogate marker of earlier-, or prodromal-, stage illness, which is likely to be more responsive to intervention (52) and (2) higher interpersonal sensitivity as potential marker of lower disorganization in psychotic illness, implying a greater capacity to participate and benefit from psychosocial treatments, such as A4i.

### Lower psychotic and obsessive compulsive traits

Conversely, participants with *lower* baseline psychotic traits and/or *lower* baseline obsessive compulsive traits were also predicted to benefit from the A4i intervention. Psychosis is often characterized by marked perceptual disturbances and disorganization, which may suggest that individuals who were more organized were able to engage more effectively and consistently with the intervention. Notably, individuals at risk of psychosis are found to experience difficulties with interpersonal relationships, manifesting as an inability to communicate distressing psychological experiences to others (53). These troubles with communication and experiential expression likely also affect patients with high levels of psychotic traits resulting in difficulties engaging with other users of A4i, and thus the intervention overall. Similarly, intervention and community engagement may have proven difficult for patients’ with obsessive compulsive traits, as these may involve intrusive, and distressing thoughts. Patients with OCD often have difficulties recognizing affective social cues, regulating emotion (54), and communicating (55). These difficulties likely also exist in individuals with primary or secondary psychoses who have obsessive compulsive traits, preventing effective engagement with socially dependent interventions, such as A4i.

### App interaction and feedback

Notably, higher baseline *depression* predicted both higher A4i engagement and more satisfaction with the app (as measured by post-intervention feedback polarity, see Figures 2B,C). This finding is consistent with research to date demonstrating the prognostic value of affective symptoms (e.g., depression) in schizophrenia (39, 56, 57). In particular, comorbid depressive symptoms have been associated with more positive outcomes, including fewer hospitalizations and fewer illness relapses in schizophrenia (39). Considered in the context of the present study results, we hypothesize depression to likewise represent a marker of ability to engage with a multi-feature digital intervention. Intuitively, it follows that individuals who had greater app engagement also had greater symptom improvement (see Figure 2A) and therefore would have greater satisfaction with the app (see Figure 2C).

In contrast to *depression*, higher *overall symptom severity* at baseline predicted lower A4i engagement and less favorable sentiment toward the app. We hypothesize individuals with greater symptom severity had more disorganization and executive functioning impairment, making it difficult to fully engage with a multi-feature digital intervention, like A4i. It follows that patients who interacted less with the app would have less symptom improvement (see Figure 2A) and therefore would have less favorable impressions (see Figure 2C). These findings are important in light of evidence suggesting overall lower digital app engagement among populations with schizophrenia, likely skewed by small subgroups of heavily engaged participants (58). Understanding the individual-level characteristics that drive engagement and sentiment toward digital technologies is essential for future mental health app development and implementation.

### Limitations

Despite the strengths of leveraging a LOO cross-validation machine learning framework to investigate unique patient markers of digital treatment response for individuals with schizophrenia, there are a number of limitations concerning the study sample and design that should be addressed. (1) The original study used a 38-person sample drawn from urban Canadian residents, limiting generalizability of the reported (2) This study did not conduct long-term patient follow-up, an important aspect of comprehensive treatment analyses, thus it is unknown whether the observed improvements in schizophrenia psychopathology will persist for these patients. As such, the long-term efficacy of A4i, and the long-term importance of the identified unique patient markers, cannot be evaluated. (3) Due to the method of analysis, the present results only reflect predictive capacity, not causality. (4) While the BSI captures positive and negative symptoms associated with psychotic disorders, structured interviews specific to psychosis and schizophrenia (e.g., PSYRATS-D) were not included. (5) The present analyses did not incorporate demographic information or lifestyle-related information, and thus cannot account for the manner in which demographic features or living and work environments influenced participant engagement with the A4i intervention.

### Conclusion

The present study sought to interrogate the A4i app as a digital intervention for schizophrenia patients *via* an ensemble LOO machine learning approach, allowing insight into the most influential, unique patient characteristics for predicting intervention response and engagement. Notably, high interpersonal sensitivity was predictive of total symptom reduction across the digital intervention, and high depression was predictive of increased digital intervention engagement and positive qualitative impressions. Taken together, these findings highlight the necessity for patient-level interrogation of treatment efficacy in mental health, particularly for schizophrenia where clinical presentation is heterogeneous. Future work should build upon the present findings to consider how individual demographic characteristics (e.g., gender, race) may influence differential engagement with a digital intervention, particularly in individuals with psychosis. Additionally, future work should aim to extend the current methodology to more traditional interventions for individuals with schizophrenia (e.g., psychosocial interventions combined with antipsychotic use) to identify unique patient characteristics predictive of intervention response, and thus tailor treatment type based on an individual’s clinical presentation.

## Data availability statement

Publicly available datasets were analyzed in this study. This data can be found here: https://journals.plos.org/plosone/article?id=10.1371/journal.pone.0219491. All relevant data are within the supporting information material.

## Ethics statement

The studies involving human participants were reviewed and approved by Research Ethics Board, Centre for Addiction and Mental Health. The patients/participants provided their written informed consent to participate in this study.

## Author contributions

GP, MH, and NJ contributed to conceptualization and design of the study. GP and MN implemented the modeling and analyses. GP, MH, MN, JM, and NJ wrote sections of the manuscript. All authors contributed to manuscript revision, read, and approved the submitted version.
