# Supplementary material for: Predicting symptom response and engagement in a digital intervention among individuals with schizophrenia and related psychoses
Source: Front Psychiatry. 2022 Aug 11;13:807116. doi: 10.3389/fpsyt.2022.807116 (PMC9403124; doi:10.3389/fpsyt.2022.807116)
Supplement: Supplementary file 1 [file Table_1.docx]

**Supplementary Table 1**

*Machine Learning Model(s) Architecture and Hyperparameters*

| *Modeling*  *Approach* | *Upper-Level Machine Learning Algorithm*  *[Hyperparameters]* | *Lower-Level Machine Learning Algorithm(s)*  *[Hyperparameters]* |
| --- | --- | --- |
| *Model 1: Symptom Severity Change* | *MLP Regressor* [alpha = 0.0001,  hidden layer sizes  = (125,200,125,100,75)] | *RandomForest Regressor* [number of estimators = 4;  max depth = 4]  *K-Neighbors Regressor* |
| *Model 2: A4i*  *Engagement* | *MLP Regressor* [alpha = 0.001,  hidden layer sizes  = (100,200,100,50,25)] | *SV Regressor* [kernel = linear]  *RandomForestRegressor* [number of estimators = 8;  max depth = 3]  *K-Neighbors Regressor*  *Lasso* [alpha = 0.01] |
| *Model 3: Intervention Impressions* | *XGBoost Regressor* | *SV Regressor* [kernel = linear]  *RandomForest Regressor*  *K-Neighbors Regressor* [number of neighbors = 4]  *XGBoost Regressor* [number of estimators = 50,  max depth = 2] |

**Supplementary Table 1.** *Ensemble model algorithms and hyperparameters used for detecting Symptom Severity Change, A4i Engagement, and Intervention Impressions, respectively. Algorithms with no hyperparameters specified did not use any user-defined hyperparameter values.*
